# Kriging-Based Land-Use Regression Models That Use Machine Learning Algorithms to Estimate the Monthly BTEX Concentration

**DOI:** 10.3390/ijerph17196956

**Published:** 2020-09-23

**Authors:** Chin-Yu Hsu, Yu-Ting Zeng, Yu-Cheng Chen, Mu-Jean Chen, Shih-Chun Candice Lung, Chih-Da Wu

**Affiliations:** 1 Department of Safety, Health and Environmental Engineering, Ming Chi University of Technology, New Taipei 243303, Taiwan; gracecyhsu@mail.mcut.edu.tw; 2 Center for Environmental Sustainability and Human Health, Ming Chi University of Technology, New Taipei 243303, Taiwan; 3 Department of Geomatics, National Cheng Kung University, Tainan 70101, Taiwan; z10708105@ncku.edu.tw; 4 National Institute of Environmental Health Sciences, National Health Research Institutes, Miaoli 35053, Taiwan; yucheng@nhri.org.tw (Y.-C.C.); zeromagi@nhri.org.tw (M.-J.C.); 5 Research Center for Environmental Changes, Academia Sinica, Taipei 11529, Taiwan; sclung@rcec.sinica.edu.tw; 6 Department of Atmospheric Sciences, National Taiwan University, Taipei 10617, Taiwan; 7 Institute of Environmental Health, National Taiwan University, Taipei 10055, Taiwan

**Keywords:** nitrogen dioxide (NO_2_), hybrid Kriging-LUR model, culture-specific sources, spatiotemporal variations

## Abstract

This paper uses machine learning to refine a Land-use Regression (LUR) model and to estimate the spatial–temporal variation in BTEX concentrations in Kaohsiung, Taiwan. Using the Taiwanese Environmental Protection Agency (EPA) data of BTEX (benzene, toluene, ethylbenzene, and xylenes) concentrations from 2015 to 2018, which includes local emission sources as a result of Asian cultural characteristics, a new LUR model is developed. The 2019 data was then used as external data to verify the reliability of the model. We used hybrid Kriging-land-use regression (Hybrid Kriging-LUR) models, geographically weighted regression (GWR), and two machine learning algorithms—random forest (RF) and extreme gradient boosting (XGBoost)—for model development. Initially, the proposed Hybrid Kriging-LUR models explained each variation in BTEX from 37% to 52%. Using machine learning algorithms (XGBoost) increased the explanatory power of the models for each BTEX, between 61% and 79%. This study compared each combination of the Hybrid Kriging-LUR model and (i) GWR, (ii) RF, and (iii) XGBoost algorithm to estimate the spatiotemporal variation in BTEX concentration. It is shown that a combination of Hybrid Kriging-LUR and the XGBoost algorithm gives better performance than other integrated methods.

## 1. Introduction

Chemical and petroleum facilities are major emitters of volatile organic compounds (VOCs) into the environment [1,2]. These industrial emissions include benzene, toluene, ethylbenzene, and xylenes, which are also known as BTEX [3,4]. On the other hand, ambient BTEX might also result from various substances and sources, including traffic, gas stations, combustion processes, and households [5,6,7]. BTEX emissions have a significant effect on human health. For example, the International Agency for Research on Cancer classifies benzene as carcinogenic for humans [8]. Benzene also affects blood production, the lymphatic system, and the central nervous system [5,9]. Even at low concentrations, BTEX has an adverse effect on reproductive processes, cardiovascular disease, respiratory dysfunction, asthma, and sensitivity to common antigens [10]. Several studies report that residents near sources of emission from chemical/petroleum facilities are exposed to relatively high levels of BTEX [11,12]. Other studies also show a positive correlation between cancer risk (leukemia and brain tumor) and exposure to benzene or surrogates for residents who live close to petrochemical facilities [12,13]. This highlights the importance of predicting BTEX concentrations to help policy makers to assess the prevention measures. However, few studies have addressed within-city variability in the level of BTEX.

Kaohsiung City, which is a heavily industrialized harbor city located in southern Taiwan, has a population of 2.8 million and an area of 153.6 km^2^. There are numerous industrial parks, petrochemical facilities, and more than 1.5 million motor vehicles in Kaohsiung. These emission sources have a negative effect on air quality in Kaohsiung, and partially on the high level of VOCs. The air quality in autumn and winter is worst because the atmosphere is stable, winds are slow, and there is diurnal land–sea breeze circulations in the cold and dry seasons. Although the Taiwanese Environmental Protection Agency (EPA) has imposed emission standards to control ambient air quality and minimize the risk to health, residents in Kaohsiung still have a long-standing concern for pollutant exposure, partly because it is still unknown how long-term VOCs exposure affects human health. To find out, the spatial variability of pollution concentration is essential.

Kriging and land-use regression (LUR) are used to predict air pollution gradients if there is limited sampled data. Kriging is a method of spatial interpolation that assumes the distance or direction between sampling points to reflect a spatial correlation. This spatial correlation is used to explain variation in the surface. The LUR model is widely used to estimate spatial variation in VOC concentrations to determine a population’s exposure to pollution [14,15,16]. In addition, using adjacent monitoring sites and local emission points as well as estimated air pollution concentrations from Kriging interpolation as a variable for LUR (Hybrid Kriging-LUR) allows a more accurate prediction of air pollution levels [17]. However, these models can ignore the dynamic spatial and temporal relationship between VOCs and predictor variables because LUR and Hybrid Kriging-LUR models only produce a single regression equation to summarize global relationships between independent and dependent variables. These regression models can also underestimate the pollution levels and fail to identify nonlinear relationships between predictions and observations that may not be linearly correlated.

A geographically weighted regression (GWR) model considers the spatial variation in relationships and creates maps to determine nonstationary spatial relationships [18]. Machine learning algorithms such as Random Forest (RF) and eXtreme Gradient Boosting (XGBoost) are also widely used to determine air pollution concentrations because they identify nonlinear relationships between observations and predictors [19,20,21].

This study used Hybrid Kriging-LUR alone and then with GWR and machine learning algorithms (RF and XGBoost) to estimate the spatiotemporal variation in BTEX in Kaohsiung. To increase the accuracy of each model, the distribution of local temples was used as a predictor to represent the unique emissions from sources that are unique to Asian culture. In terms of residents’ health and indicators of the health effect, this study shows that air epidemiological studies of ambient BTEX are important for the future.

## 2. Materials and Methods 

### 2.1. Study Area

Kaohsiung is an industrial city located in southern Taiwan, with a population density of 3957 (people per km^2^), three petrochemical industrial parks, a large iron ore and steel factory, and many factories that use oil/coal combustion. There are about 3 million registered motor vehicles (including motorbikes, cars, and other vehicles) in this city. There are 72 vehicles per hundred people so traffic emissions are a significant factor for air pollution in Kaohsiung. On average there are 10 factories per square kilometer and many of these are located near commercial districts and residential areas. Local culture also plays a role in this study because Taiwan features unique emission sources of BTEX, such as the frequent burning of joss paper and incense in thousands of temples [22,23]. The present study area crossed six districts in Kaohsiung and one district in Pingung (Figure S1). It also covered two large petrochemical industrial parks (Linhai and Linyuan). The study districts contributed respective ~50% of sulfur oxides (SOx), ~60% of nitrogen oxides (NOx), and ~60% of VOCs to ambient pollutants in Kaohsiung in 2018. The registered vehicles in the study districts were about 40% of total in Kaohsiung. In addition, the largest iron ore and steel factory in Taiwan is located in Linhai petrochemical industrial park.

### 2.2. Air Pollutant Database

The Taiwanese EPA requires that air pollution monitoring stations must be established in all townships in close proximity to petrochemical industrial parks. This study uses five years of BTEX data (from May 2015 to June 2019) collected from 17 monitoring stations that are close to two petrochemical industrial parks in south Kaohsiung (shown in Figure S1). The data from 2015 and 2018 were used to develop models and observations from 2019 and are also used for external data verification to assess the reliability of the models.

This study uses 10,660 hourly measurements of BTEX, which are aggregated into 939 monthly averages for the model. The concentrations of monitored pollutants are obtained from the EPA database as explanatory variables. Previous studies confirm monitored pollutants’ association with BTEX concentration (e.g., NOx and O_3_) [24,25]. Table S1 lists the potential predictor variables used in this study.

### 2.3. Geospatial Database

To develop Hybrid Kriging-LUR, information about land-use or land-cover from several GIS layers and spatial databases is required. The land-use inventory, the landmark database, the digital road network map, the Digital Terrain Model (DTM), Moderate Resolution Imaging Spectroradiometer (MODIS) Normalized Difference Vegetation Index (NDVI) database, and the thermal power plant distribution dataset are used. Further details of land-use or land-cover related information for potential prediction variables (Table S1) can be found in a previous study by the authors [17,26,27]. In this study, LUR models at monthly resolution were developed based on air pollutants measurements from 2015 to 2019. It is difficult to obtain clear Landsat or SPOT images for every month in Taiwan due to the humid and cloudy weather [28]. In this case, we just followed previous studies to obtain NDVI information from MODIS [29].

### 2.4. LUR Model Development and Validation

This study uses a Hybrid Kriging-LUR to identify important prediction variables. The variables that are selected by Hybrid Kriging-LUR are used for the geographically weighted regression (GWR) and two machine learning algorithms (random forest (RF) and extreme gradient boosting (XGBoost)) to develop the prediction models called RF-Hybrid LUR or XGBoost-Hybrid LUR.

This study uses the same Hybrid Kriging-LUR as that proposed by Wu et al. [17]. Kriging-based estimations of the BTEX level are used as an explanatory variable for a stepwise selection during the conventional LUR procedure, using a leave-one-out Ordinary Kriging interpolation algorithm for “n-1” observations. The Hybrid Kriging-LUR approach uses Spearman correlation coefficients to define the bivariate association between each BTEX compound and all of the potential prediction variables. A supervised stepwise procedure is used to maximize the percentage of explained variability (R^2^). For all potential predictor variables, an a-priori direction of effect is used for each BTEX (e.g., positive for road length and residential area to benzene). The variable initially has the highest explained variance in a univariate analysis and a regression slope with the expected direction. All other variables are then added to this model separately by assessing whether the *p*-value is <0.1 and the variance inflation factor (VIF) is <3. This procedure continues until none of the variables fit the specified criteria. Finally, The R^2^ adjusted R^2^ values and the Root Mean Square Error (RMSE) are used to determine the model’s performance.

GWR is used to solve the model spatially. The equation (Equation (1)) for the GWR model is defined as follows:

(1) $$Y_{i}{=\beta }_{\left(U_{i}{,v}_{i}\right)}+\sum_{k}\beta_{k\left(U_{i}{,v}_{i}\right)}X_{\mathrm{ik}},$$

where ($U_{i}{,v}_{i}$) denotes the coordinates of the point in a location; $Y_{i}$ is BTEX concentration; $\beta_{\left(U_{i}{,v}_{i}\right)}$ represents the intercept value; $\beta_{k\left(U_{i}{,v}_{i}\right)}$ is a set of values of parameters at point i; and $X_{\mathrm{ik}}$ are prediction variables that are obtained using Hybrid Kriging-LUR approaches.

The RF grows multiple decision trees and forces a randomly selected subset of candidate predictors into each tree [30]. RF-based Hybrid Kriging-LUR approaches produce 200 regression trees, which are extracted from randomly bootstrapped features from the training data. The extent to which a tree grows also affects the model’s performance. This study uses depths of 30 for Hybrid Kriging-LUR models.

XGBoost is a common machine learning algorithm that was first proposed by Chen and Guestrin [31]. It has been proved very successful in many machine learning competitions. XGBoost is similar to a random forest approach in that it features multiple regression trees. The tree ensemble model trains weak learners to optimize the model using the bias for the loss function by boosting a scalable gradient tree. If XGBoost learners with a different feature importance score are generated across all trees, the prediction is accumulated in terms of the weight of each learner. The Hybrid Kriging-LUR approaches use 130 trees, and the maximum depths to which the trees are grown was 8. The parameter values for each method are listed in Tables S2 and S3.

Land-use/land-cover information is extracted using ArcGIS 10.5 (Esri, Redlands, CA, USA). LUR and all statistical analyses are conducted using SPSS 22.0 (IBM, New York, NY, USA) and R 3.5.2. (The R Foundation for Statistical Computing, Vienna, Austria) These machine learning models are programmed in Python 3.7, using a Jupyter Notebook platform. The computer hardware is a laptop (ASUS, Taipei City, Taiwan) with a CPU i5-8265U and 8 GB of RAM.

## 3. Results

### 3.1. Descriptive Statistics for BTEX Concentrations

Figure S1 shows annual wind rose for 2015–2018. The site experiences a predominantly Westerly wind flow in the spring and winter, in all directions in the summer, and a Westerly to Northern wind flow in the fall. The winds generally blew at 0.2 to 5.37 m/s and at an average 2.3 ± 1.35 m/s. In terms of annual BTEX concentration, toluene is the dominant BTEX compound in the study area (3.31 ± 4.01 ppb), followed by benzene (1.22 ± 5.57 ppb), m,p-xylene (0.78 ± 1.46 ppb), and ethylbenzene (0.49 ± 1.16 ppb). The highest BTEX concentrations in Kaohsiung were greater than those in Beijing (1.44 ppb for toluene, 0.54 ppb for benzene, 0.48 ppb for m,p-xylene, and 0.27 ppb for ethylbenzene) [32] and Tianjin (0.50 ppb for toluene, 1.22 ppb for benzene, 0.57 ppb for m,p-xylene, and 0.51 ppb for ethylbenzene) [33] in China. Kaohsiung also has higher BTEX figures than areas near the largest petrochemical industrial parks in Taiwan (2.56 ppb for toluene, 0.22 ppb for benzene, 0.14 ppb for m,p-xylene, and 0.07 ppb for ethylbenzene) [34].

**Figure 1:**
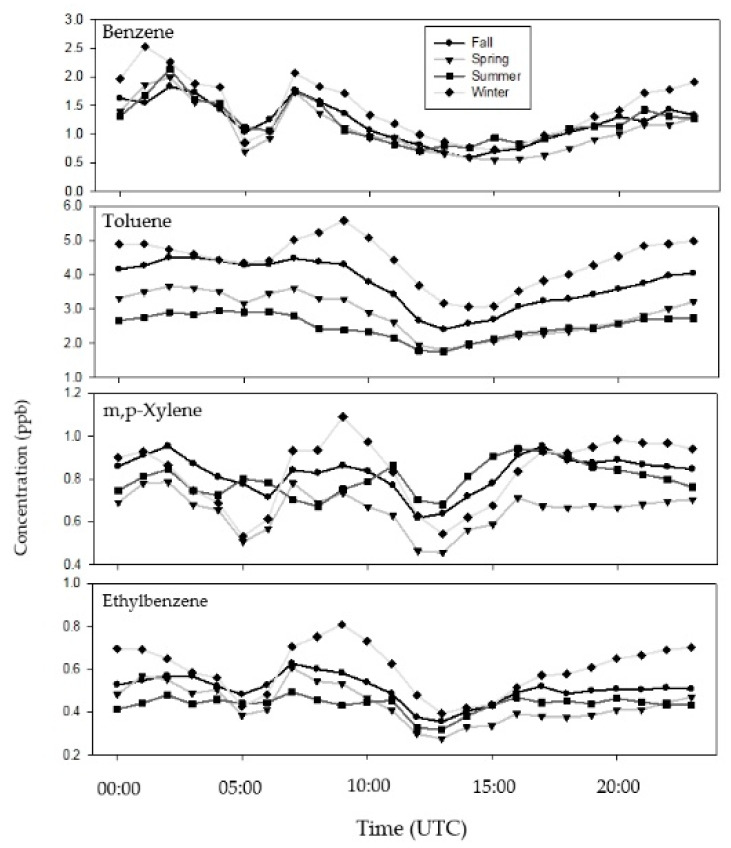
The diurnal variation in the BTEX (benzene, toluene, ethylbenzene, and xylenes) concentrations for each season, averaged over 17 sampling sites.

Figure 1 shows BTEX average diurnal variations in each season during the study period. For example, the concentration of BTEX compounds became relatively lower in the daytime (from 10:00 to 15:00) with the lowest concentration observed at ~13:00. This is consistent with what has been reported in Shanghai for a similarly-situated large industrial estate [35]. Such a diurnal trend is likely caused by strong solar radiation and intense air convection in the daytime, both of which can photochemically react with and/or dilute VOCs [35]. In contrast, as shown in Figure 1, we can see higher BTEX concentrations during rush hours both in the morning (7:00 to 9:00) and late afternoon (~18:00), similar to the findings in previous studies [36,37]. Such a high concentration also suggests that automobile exhaustion was an important source for atmospheric BTEX in the study area. Figure 1 further shows similar diurnal variations of BTEX across four seasons, indicating that the BTEX concentration is contributed from similar sources and dispersion mechanisms in each season. In addition, some variances of BTEX concentrations in Figure 1 are likely influenced by many factors such as emission sources (mainly from vehicular exhaust, gasoline, and solvent evaporation), meteorological conditions, and their sinks, given that the study area is located in the industrial area of Kaohsiung. Indeed, benzene, toluene, and xylenes are typical tracers of vehicular exhaust, industrial production, and solvent usage, respectively [38,39]. This also explains why Figure 1 shows distinct diurnal variations of BTEX concentration in each season.

### 3.2. Development and Validation of The LUR and Machine Learning Models

Table 1 shows the selected prediction variables, the estimated coefficient, the partial R^2^ value and the VIF for the proposed Hybrid Kriging-LUR model. The variables, Benzene_Kriging-based_, UV, rice farm within a 150-m buffer, and harbor and industrial area within a 500-m buffer, all have a significant effect on the explanatory power of the model for benzene. In terms of toluene, the significant variables are toluene_Kriging-based_, NO_x_, water body, purely residential area within a 250-m buffer, sandstone field within a 150-m buffer, sandstone field within a 2500-m buffer, industrial area within a 150-m buffer, all types of road within a 50-m buffer, and temple within a 250-m buffer. In terms of ethylbenzene, the significant variables are ethylbenze_Kriging-based_, SO_2_, winter, industrial area within a 250-m buffer, temple within a 250-m buffer, fruit orchard within a 50-m buffer, and fruit orchard within a 1500-m buffer. In terms of m,p-xylene, the factors that have the most significant effect on the explanatory power of the model are m,p-xylene_Kriging-based_, sandstone field within a 150-m buffer, funeral services within a 1250-m buffer, industrial area within a 50-m buffer, local road within a 250-m buffer, and temple within a 250-m buffer. All of these variables discussed are used to develop hybrid models, such as GWR with the Hybrid Kriging-LUR (GWR-Hybrid LUR), RF with the Hybrid Kriging-LUR (RF-Hybrid LUR), and XGBoost with the Hybrid Kriging-LUR (XGBoost-Hybrid LUR). Most variables have a positive effect on BTEX, except for UV and harbor for benzene, and sandstone field for toluene, ethylbenzene, and m,p-xylene.

Table 2 shows the performance of the Hybrid Kriging-LUR, GWR-Hybrid LUR, RF-Hybrid LUR, and XGBoost-Hybrid LUR models. The XGBoost-Hybrid LUR better predicts the variation in all BTEXs, with a R^2^ value from 0.61 to 0.79. The Hybrid Kriging-LUR has the worst R^2^ value (from 0.37 to 0.52). Similar results as to R^2^ were obtained in adjusted R^2^ values (from 60 to 79 for the XGBoost-Hybrid LUR, which performs best; and 0.37 to 0.52 for the Hybrid Kriging-LUR, which performs worst). 

The XGBoost-Hybrid LUR has the lowest RMSE (from 0.24 ppb to 1.03 ppb) and the Hybrid Kriging-LUR has the highest RMSE (from 0.31 ppb to 1.1.35 ppb). The adjusted R^2^ values are similar for the overfitting tests (Table 2). The respective adjusted R^2^ values for testing for the Hybrid Kriging-LUR, the GWR-Hybrid LUR, RF-Hybrid LUR, and the XGBoost-Hybrid LUR models are 0.34–0.56, 0.22–0.59, 0.38–0.77, and 0.50–0.79.

Observations from January to June in 2019 were used as external data to verify the robustness of the model (Table 3). The respective adjusted R^2^ values for the Hybrid Kriging-LUR, GWR-Hybrid LUR, the RF-Hybrid LUR, and the XGBoost-Hybrid LUR models are 0.34–0.65, 0.28–0.58, 0.42–0.56, and 0.41–0.55. This shows that even if the R^2^ value is reduced, these models still have a medium level of prediction performance. To validate the exposure estimates, we further conducted a 10-fold cross-validation to verify the model performance of the XGBoost-Hybrid LUR. 90% of the sites’ data were randomly selected for model development, while the remaining 10% were used as out-of-data for model evaluation. This procedure was repeated ten times; thus, each monitoring site was used as a test data set for spatial verification. Similar R^2^ values with the main model (0.53 for benzene, 0.56 for toluene, 0.48 for ethylbenzene, and 0.59 m,p-xylene in Table S4) were obtained again to confirm the reliability of the developed model (0.41 for benzene, 0.55 for toluene, 0.45 for ethylbenzene, and 0.52 m,p-xylene in Table 3).

### 3.3. Spatiotemporal Distribution of BTEX

**Figure 2:**
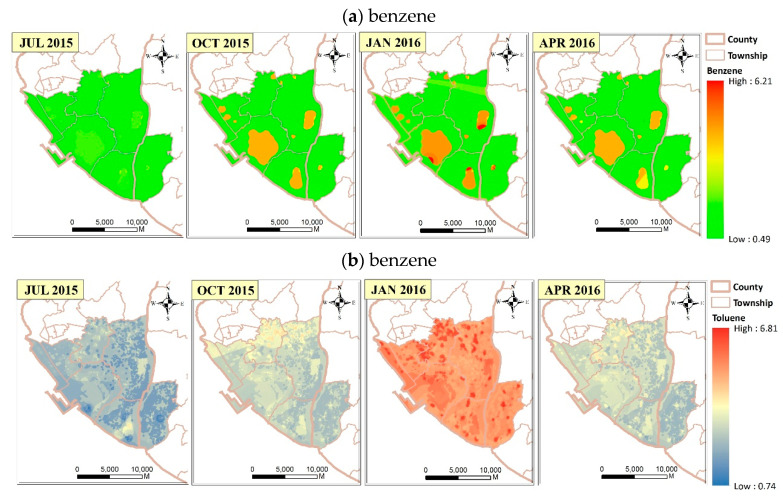

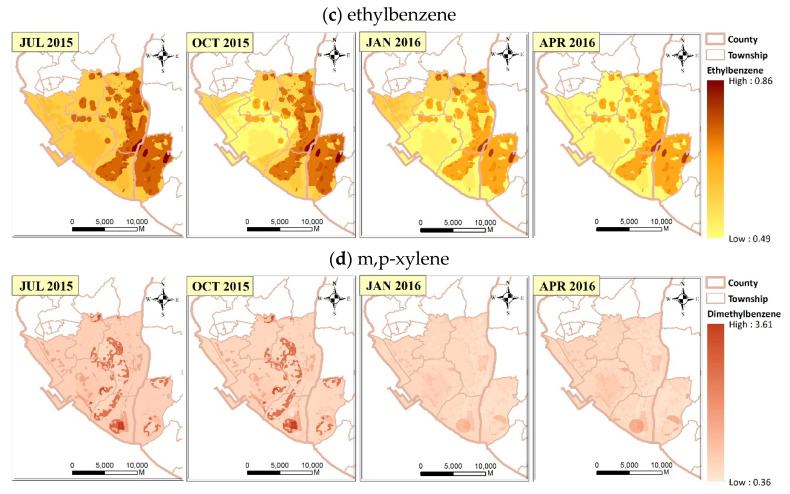
Monthly average concentration of BTEX: (**a**) benzene, (**b**) toluene, (**c**) ethylbenzene, and (**d**) m,p-xylene.

By using the XGBoost-Hybrid LUR model for representative months from 2015 to 2016 (July, October, January, and April), Figure 2 shows the monthly average BTEX concentration through the study period. To begin with, the spatial variation in each season was relatively consistent, probably because the season and temperature factors were too insignificant to be selected into the models (Table 1). Second, there are higher benzene concentrations (light yellow to red color in Figure 2a) near industrial parks because of the higher partial R^2^ for the factor of industry in the benzene model (Table 1). Third, as shown in Figure 2b, we see higher toluene levels scattering in places closer to the city or roads (red color in Jan 2010 in Figure 2b) because NOx, which is the major pollutant of traffic, had higher partial R^2^ in the toluene model (Table 1). Fourth, higher ethylbenzene concentrations (dark brown color in Figure 2c) were shown in certain residential areas with many temples and near industrial parks because of the higher partial R^2^ for the factor of both industry and temples in the ethylbenzene model. Fifth, higher m,p-xylene concentrations (dark brown color in Figure 2d) were seen also in certain residential areas with many temples because of the higher partial R^2^ for the factor of temples in the m,p-xylene model.

## 4. Discussion

Most studies of exposure to ambient BTEX and health outcomes rely on daily monitoring of air pollution [40]. Few studies determine individual exposure levels using spatial analysis techniques [14,41,42]. These studies extrapolate actual measurements to individual exposures, so they do not reliably reflect the effect of air pollutants on health outcomes. This study proposes a method that is more economical than daily monitoring and more accurate than extrapolation to determine the effect of BTEX on health.

Four spatiotemporal models are used to predict monthly average BTEX concentration from 2015 to 2018 at a resolution of 50 × 50 m. The models that combine Hybrid Kriging-LUR with machine learning (RF-Hybrid LUR and XGBoost-Hybrid LUR) have a greater predictive ability than the two regression models (Hybrid Kriging-LUR and GWR-Hybrid LUR). Specifically, the use of machine learning models in conjunction with land-use information increases the predictive power by 16% to 25% over that of the regression models. This increase is attributable to the fact that both the RF and XGBoost methods identify potential nonlinear associations between candidate predictors and ambient BTEX. To the authors’ best knowledge, this is the first study to compare machine learning and standard linear regression models to predict spatial differences in ambient BTEX. It is shown that both machine learning models (RF and XGBoost method) have a greater predictive power than standard approaches. 

XGBoost-Hybrid LUR is demonstrated to be the best model in this study and better explains the spatial variation in ambient BTEX in south Kaohsiung. The model also performs acceptably when verified using an external dataset. To address the problem of overfitting, 80% of data was used to train the XGBoost-Hybrid LUR model and 20% to test it. The adjusted R^2^ values for training and testing are similar to those for the original model so there is no overfitting problem.

The variables were selected using the Hybrid Kriging-LUR model and then used for the other three models developed by this study. Most of the significant variables are similar for the prediction of BTEX using the Hybrid Kriging-LUR model, but the individual contribution of each variable to the models is different. For example, while industrial area is a significant variable in predicting benzene, toluene, ethylbenzene, and m,p-xylenes, the significance of this variable (24%, 3%, 16%, and 8%, respectively) is different for each model (Table 1). This difference is probably caused by different levels of emissions of various compounds in each industrial area. It also highlights the need to consider different air pollutants when developing a prediction model [43]. 

When compared to other LUR models developed earlier in Sarnia, Ontario, Canada [43]; Toronto, Canada [44]; Dallas, Texas, USA [42]; Tehran, Iran [14]; Detroit/Dearborn, USA [45]; and New York City, USA [46]; this study selected both similar and different significant variables. For instance, Atari and Luginaah [43] reported that industrial area was the most significant factor for BTEX levels, while Smith et al. [42], Su et al. [44], and Amini et al. [14] suggested that traffic was the dominant factor for BTEX concentration. Mukerjee et al. [45] noted that both traffic and emission sources caused higher concentrations of BTEX. For this study, industrial area is the most significant factor for benzene and ethylbenzene. Table 1 also shows that traffic is the dominant factor for toluene concentration because 50% of NO_x_ across Taiwan and 85% of NO_x_ in cities is emitted from vehicles [47]. Sources of emissions that are specific to Asian culture, such as temples, are the dominant factor for m,p-xylene level because incense combustion significantly increases the concentration of m,p-xylene [48]. As strong solar radiation removes VOCs through the photochemical reactions [35], UV is a significant variable for the prediction of benzene. It is noteworthy that some greenness, such as rice farms and fruit orchards, can also increase BTEX levels. 

While industry and traffic are often the dominant factors in the prediction of BTEX, some BTEX sources are specific to Asia. Going to a temple to pray and burn incense and joss paper is an important religious activity for many Asian households [49], and several studies have shown that this activity contributes to air pollution [50,51,52]. However, none of these used culturally specific variables to develop an LUR model to predict BTEX. This study uses the number of temples to reflect local emissions caused by the burning of joss paper and incense, which is a significant predictor for the proposed model. Future studies should also consider this unique local cultural source as a predictor of BTEX for developing LUR models in other Asian regions.

There are limitations to the selected predictors for this study. Traffic intensity is used by other studies to improve model performance [45,53] but is not used for this study because data are not generally available in Taiwan. In stand, we used NOx as proxies for traffic because a great portion (50% to 80%) of NO_x_ is emitted from vehicles in Taiwan [47]. In contrast to data for a single year or an even shorter period, which is used by other studies, this study uses a much longer period (from 2015 to 2019) to represent spatial and temporal variations in compound concentrations. Using long-term pollutant data to establish an LUR model that is refined by machine learning and considering culturally specific predictors, this model has good prediction performance, which can be used to better depict the variation of BTEX in Asian cities.

## 5. Conclusions

Using machine learning algorithms to estimate individual levels of ambient air pollution is common practice. Combining a traditional LUR model and machine learning, this study develops Hybrid Kriging-LUR, GWR-Hybrid LUR, RF-Hybrid LUR, and XGBoost-Hybrid LUR models to predict BTEX concentrations. The study site is in Kaohsiung, Taiwan, where traffic, industrial area, and temple are the main variables. Using data from seventeen measurement stations, this study shows that the machine learning LUR models (such as RF-Hybrid LUR and XGBoost-Hybrid LUR models) can better estimate fine spatial variability in long-term BTEX concentrations. This approach should be used in future studies to develop hybrid LUR models for other pollutants in Taiwan. In terms of residents’ health or health effect indicators, the results of this study support the need for future air epidemiological studies of ambient BTEX.

## Figures and Tables

**Table 1:** Prediction variables for the Hybrid Kriging-LUR model. LUR—land-use regression; VIF—variance inflation factor.

BTEX	Variable	Coefficient	*p*-Value	Partial R^2^	VIF
Benzene	Intercept	1.964	<0.05	-	-
	Benzene_Kriging-based_	0.223	<0.05	0.006	1.395
	Ultraviolet	−0.163	<0.05	0.045	1.394
	Rice farm_150m_	0.002	<0.05	0.068	1.272
	Harbor_Nearest distance_	−1.113 × 10^−4^	<0.05	0.070	1.163
	Industry_500m_	0.002	<0.05	0.240	1.185
Toluene	Intercept	−1.229	<0.05	-	-
	Toluene_Kriging-based_	0.581	<0.05	0.061	2.366
	Nitrogen Oxides	0.068	<0.05	0.246	2.311
	Water body_Nearest distance_	5.966 × 10^−4^	<0.05	0.001	1.412
	Purely residential area_250m_	0.002	<0.05	0.048	1.649
	Sandstone field_150m_	−0.005	<0.05	0.058	1.102
	Sandstone field _2500m_	0.002	<0.05	0.002	1.257
	Industry_150m_	6.208 × 10^−4^	<0.05	0.025	1.406
	All types of road(width)_50m_	3.241 × 10^−4^	0.153	0.005	1.359
	Temple_250m_	0.515	<0.05	0.071	1.403
Ethylbenzene	Intercept	−0.105	0.442	-	-
	Ethylbenze_Kriging-based_	0.072	0.239	0.007	1.097
	SO_2_	0.094	<0.05	0.032	1.342
	winter	0.114	<0.05	0.011	1.299
	Industry_250m_	3.737 × 10^−4^	<0.05	0.160	1.072
	Temple_250m_	0.105	<0.05	0.096	1.056
	Sandstone field _500m_	−3.224 × 10^−4^	<0.05	0.010	1.928
	Fruit orchard_50m_	6.428 × 10^−4^	<0.05	0.038	1.635
	Fruit orchard_1500m_	5.927 × 10^−4^	0.161	0.003	2.434
m,p-Xylene	Intercept	−0.045	0.778	-	-
	m,p-Xylene_Kriging-based_	0.432	<0.05	0.041	1.062
	Sandstone field _150m_	−8.339 × 10^−4^	0.079	0.040	1.169
	Funerary services_1250m_	0.003	<0.05	0.011	1.041
	Industry_50m_	6.963 × 10^−4^	<0.05	0.075	1.516
	Local road_250m_	16.121	<0.05	0.010	1.518
	Temple_250m_	0.364	<0.05	0.248	1.042

BTEX = benzene, toluene, ethylbenzene, and xylenes.

**Table 2:** Performance of the Hybrid Kriging-LUR, GWR-Hybrid LUR, RF-Hybrid LUR and XGBoost- Hybrid LUR models. GWR—geographically weighted regression; LUR—Land-use regression; RF—random forest; XGBoost—extreme gradient boosting; RMSE—root mean square error.

BTEX	Statistic	Hybrid Kriging-LUR	GWR-Hybrid LUR	RF-Hybrid LUR	XGBoost-Hybrid LUR
Benzene	R^2^ (training, testing)	0.45 (0.43, 0.55)	0.47 (0.46, 0.45)	0.57 (0.59, 0.42)	0.63 (0.65, 0.53)
	Adjusted R^2^ (training, testing)	0.45 (0.42, 0.54)	0.47 (0.46, 0.44)	0.56 (0.59, 0.38)	0.63 (0.64, 0.50)
	RMSE (training, testing)	1.24 (1.29, 1.06)	1.22 (1.23, 0.44)	1.10 (1.11, 1.04)	1.02 (1.01, 1.03)
Toluene	R^2^ (training, testing)	0.52 (0.52, 0.56)	0.54 (0.52, 0.60)	0.69 (0.70, 0.63)	0.72 (0.74, 0.60)
	Adjusted R^2^ (training, testing)	0.52 (0.51, 0.56)	0.54 (0.52, 0.59)	0.68 (0.69, 0.59)	0.71 (0.73, 0.56)
	RMSE (training, testing)	1.35 (1.42, 1.10)	1.33 (1.32, 1.36)	1.09 (1.07, 1.16)	1.03 (1.03, 1.16)
Ethylbenzene	R^2^ (training, testing)	0.37 (0.36, 0.49)	0.38 (0.31, 0.23)	0.50 (0.50, 0.45)	0.61 (0.62, 0.54)
	Adjusted R^2^ (training, testing)	0.37 (0.34, 0.49)	0.38 (0.31, 0.22)	0.49 (0.49, 0.40)	0.61 (0.61, 0.50)
	RMSE (training, testing)	0.31 (0.33, 0.23)	0.31 (0.32, 0.17)	0.28 (0.29, 0.22)	0.60 (0.25, 0.22)
m,p-Xylene	R^2^ (training, testing)	0.42 (0.42, 0.43)	0.44 (0.40, 0.29)	0.77 (0.77, 0.77)	0.79 (0.79, 0.79)
	Adjusted R^2^ (training, testing)	0.42 (0.41, 0.42)	0.44 (0.40, 0.29)	0.77 (0.77, 0.77)	0.79 (0.79, 0.77)
	RMSE (training, testing)	0.70 (0.72, 0.67)	0.69 (0.72, 0.27)	0.44 (0.41, 0.44)	0.42 (0.36, 0.61)

**Table 3:** External data validation for the proposed models.

BTEX	Statistic	Hybrid Kriging-LUR	GWR-Hybrid LUR	RF-Hybrid LUR	XGBoost-Hybrid LUR
Benzene	R^2^	0.52	0.52	0.44	0.41
	Adjusted R^2^	0.52	0.52	0.43	0.40
	RMSE	0.29	0.29	0.31	0.80
Toluene	R^2^	0.65	0.58	0.56	0.55
	Adjusted R^2^	0.64	0.58	0.55	0.54
	RMSE	0.81	0.88	0.90	0.91
Ethylbenzene	R^2^	0.47	0.43	0.42	0.45
	Adjusted R^2^	0.47	0.42	0.41	0.44
	RMSE	0.15	0.16	0.16	0.16
m,p-Xylene	R^2^	0.34	0.28	0.51	0.52
	Adjusted R^2^	0.34	0.27	0.51	0.52
	RMSE	0.24	0.25	0.23	0.19

## Supplementary Materials

The following are available online at https://www.mdpi.com/1660-4601/17/19/6956/s1, Figure S1: Overview of the sampling sites (1–17) and wind rose diagrams for the study periods of spring, summer, fall and winter, Table S1: Parameters proposed in hybrid-kriging LUR coupled with RF models, Table S2: Parameters proposed in hybrid-kriging LUR coupled with XGBoost models, Table S3: Parameters proposed in hybrid-kriging LUR coupled with XGBoost models, Table S4: Results of 10-fold cross-validation proposed in the XGBoost- Hybrid LUR model.
