# Supplementary material for: Kriging-Based Land-Use Regression Models That Use Machine Learning Algorithms to Estimate the Monthly BTEX Concentration
Source: Int J Environ Res Public Health. 2020 Sep 23;17(19):6956. doi: 10.3390/ijerph17196956 (PMC7579284; doi:10.3390/ijerph17196956)
Supplement: Supplementary file 1 [file ijerph-17-06956-s001.pdf]

Supplementary material

Kriging-Based Land-Use Regression Models that Use Machine Learning Algorithms to Estimate the Monthly BTEX Concentration

Table S1. List of potential predictor variables.

Database	Period	Variable	Unit
Land-use Inventory	2015	Pure residential area<br>Residential area mixed with industrial area<br>Residential area mixed with commercial area<br>Mixed residential area<br>All residential area<br>Rice farm<br>Rain fed crop<br>Fruit orchard<br>Mixed crop<br>Forest<br>Water body<br>Park<br>Industry<br>Funerary services<br>Railway<br>Sandstone field<br>Port<br>International airport	Density [Area (m^2) / Buffers Radius [a] (m)] 、Nearest Distance (m)
Map of Industrial Park	2015	Industrial park	Density [Area (m^2) / Buffers Radius (m)] 、Nearest Distance (m)
Digital Road Network Map	2015; 2017	Local road<br>Major road<br>All road	Density [Area (m^2) / Buffers Radius (m) 、Length (m) / Buffers Radius (m)]
POI Landmark Database	2010	Temple<br>Chinese restaurant<br>Temple and Chinese restaurant<br>Funeral facility<br>Cemetery and crematorium	Density [Count / Buffers Radius (m)] 、Nearest Distance (m)
Power Plant	2017	Thermal power plant	Nearest Distance (m)
EPA Incineration Plants	2014	Incinerator stack	Nearest Distance (m)
Digital Terrain Model	2015	Altitude	Elevation (m)
Global MODIS NDVI	2015-2019	Average NDVI<br>Maximum NDVI<br>Minimum NDVI	Monthly Average
EPA Air Pollutant Monitoring Database	2015-2019	NO_2<br>NO_X<br>O_3<br>SO_2	Monthly Average (ppb)
		PM_{10}<br>$PM_{2.5}$	Monthly Average (μg/m^3)
		CO	Monthly Average (ppm)
CWB Weather Database	2015-2019	Temperature	Monthly Average (℃)
		Relative humidity	Monthly Average (%)
		Atmospheric pressure	Monthly Average (hPa)

		Rainfall<br>UV<br>Wind speed<br>Wind direction	Monthly Summation (mm)<br>Monthly Average<br>Monthly Average (km/hour)<br>Monthly Average (°)
Dummy Variables	2015-2019	Spring<br>Summer<br>Fall<br>Winter	1 or 0

Table 2. Parameters proposed in hybrid-kriging LUR coupled with RF models.

BTEX	Estimators	Min samples leaf	Max depth	Criterion
Benzene	100	11	10	mse
Toluene	100	4	10	mse
Ethylbenzene	140	6	10	mse
m,p-Xylene	50	1	3	mse

Table 3. Parameters proposed in hybrid-kriging LUR coupled with XGBoost models.

BTEX	Learning rate	Alpha	Estimators	Max depth
Benzene	0.1	0	36	2
Toluene	0.1	0	49	3
Ethylbenzene	0.1	0	20	3
m,p-Xylene	0.1	0	75	2

Table 4. Results of 10-fold cross-validation proposed in the XGBoost- Hybrid LUR model.

BTEX	Folder	R^2	Total R^2
Benzene	1	0.24	Mean 0.51<br>Median 0.53
	2	0.25	
	3	0.45	
	4	0.46	
	5	0.50	
	6	0.56	
	7	0.56	
	8	0.59	
	9	0.61	
	10	0.91	
Toluene	1	0.27	Mean 0.513<br>Median 0.56
	2	0.45	
	3	0.50	
	4	0.53	
	5	0.54	
	6	0.58	
	7	0.60	
	8	0.60	
	9	0.62	
	10	0.63	
Ethylene	1	0.02	Mean 0.47<br>Median 0.48
	2	0.33	
	3	0.38	
	4	0.44	
	5	0.46	
	6	0.50	
	7	0.55	
	8	0.55	
	9	0.67	
	10	0.76	
	1	0.41	
	2	0.42	
	3	0.46	
	4	0.46	Mean 0.59

m,p-Xylene	5	0.58	Median 0.59
	6	0.60	
	7	0.66	
	8	0.73	
	9	0.79	
	10	0.80	

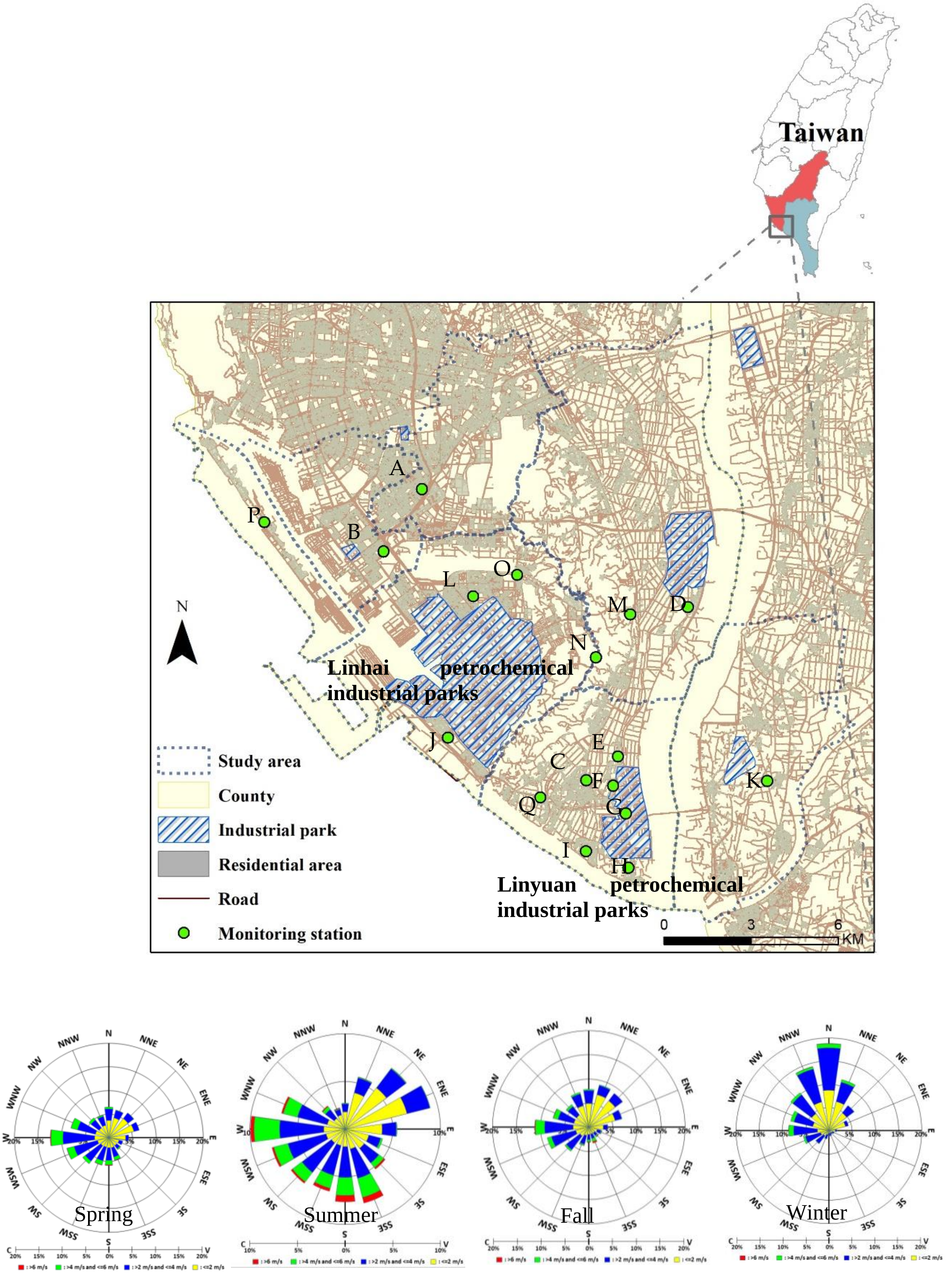

Figure 1. Overview of the sampling sites, including urban (site A-C), industrial areas (site D-K on Fig. S1), and rural areas (site L-Q on Fig. S1), and wind rose diagrams for the study periods of spring, summer, fall and winter.
